# Supplementary material for: Screening the Significant Hub Genes by Comparing Tumor Cells, Normoxic and Hypoxic Glioblastoma Stem-like Cell Lines Using Co-Expression Analysis in Glioblastoma
Source: Genes (Basel). 2022 Mar 15;13(3):518. doi: 10.3390/genes13030518 (PMC8951270; doi:10.3390/genes13030518)
Supplement: Supplementary file 1 [file genes-13-00518-s001.zip › genes-1614549-supplementary.pdf]

Supplementary Materials

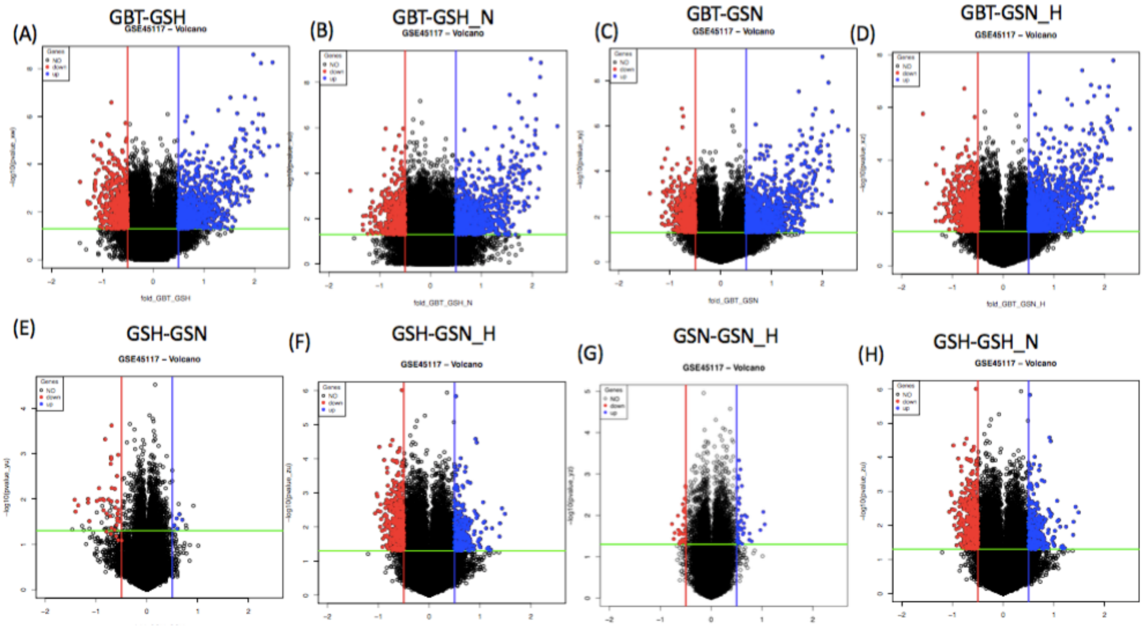

Figure S1. Volcano plots of each paired treatments of DEGs of GSE45117 gene expression dataset.

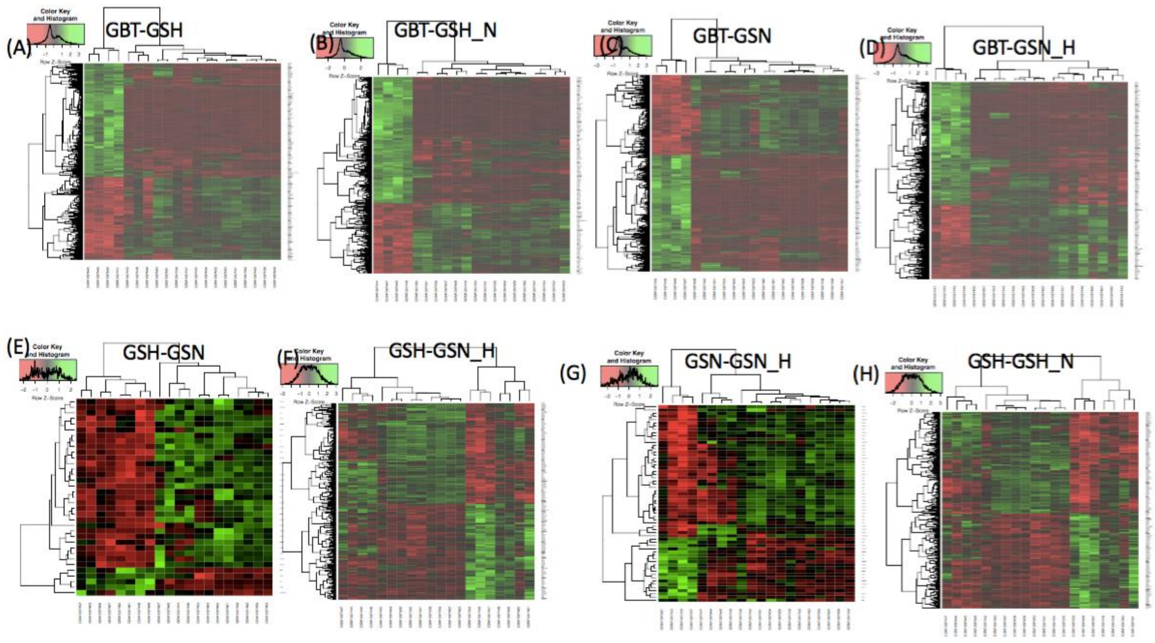

Figure S2. Heatmap plots of each paired treatments of DEGs of GSE45117 gene expression dataset.

Table S1. https://www.dropbox.com/s/14n53c91apnw38p/Table%20S1.csv?dl=0
